# Structural landscape of the complete genomes of dengue virus serotypes and other viral hemorrhagic fevers

**DOI:** 10.1186/s12864-021-07638-7

**Published:** 2021-05-17

**Authors:** Riccardo Delli Ponti, Marek Mutwil

**Affiliations:** School of Biological Sciences, Nanyang Technological University, 60 Nanyang Drive, Singapore, 637551 Singapore

**Keywords:** Genome structure, Secondary structure, Virus

## Abstract

**Background:**

With more than 300 million potentially infected people every year, and with the expanded habitat of mosquitoes due to climate change, Dengue virus (DENV) cannot be considered anymore only a tropical disease. The RNA secondary structure is a functional characteristic of RNA viruses, and together with the accumulated high-throughput sequencing data could provide general insights towards understanding virus biology. Here, we profiled the RNA secondary structure of > 7000 complete viral genomes from 11 different species focusing on viral hemorrhagic fevers, including DENV serotypes, EBOV, and YFV.

**Results:**

In our work we demonstrated that the secondary structure and presence of protein-binding domains in the genomes can be used as intrinsic signature to further classify the viruses. With our predictive approach, we achieved high prediction scores of the secondary structure (AUC up to 0.85 with experimental data), and computed consensus secondary structure profiles using hundreds of in silico models. We observed that viruses show different structural patterns, where e.g., DENV-2 and Ebola virus tend to be less structured than the other viruses. Furthermore, we observed virus-specific correlations between secondary structure and the number of interaction sites with human proteins, reaching a correlation of 0.89 in the case of Zika virus. We also identified that helicases-encoding regions are more structured in several flaviviruses, while the regions encoding for the contact proteins exhibit virus-specific clusters in terms of RNA structure and potential protein-RNA interactions. We also used structural data to study the geographical distribution of DENV, finding a significant difference between DENV-3 from Asia and South-America, where the structure is also driving the clustering more than sequence identity, which could imply different evolutionary routes of this subtype.

**Conclusions:**

Our massive computational analysis provided novel results regarding the secondary structure and the interaction with human proteins, not only for DENV serotypes, but also for other flaviviruses and viral hemorrhagic fevers-associated viruses. We showed how the RNA secondary structure can be used to categorise viruses, and even to further classify them based on the interaction with proteins. We envision that these approaches can be used to further classify and characterise these complex viruses.

**Supplementary Information:**

The online version contains supplementary material available at 10.1186/s12864-021-07638-7.

## Background

Dengue virus (DENV) is a mosquito-borne virus that can potentially infect more than 300 million people a year in more than 120 countries [1, 2]. DENV infection can further evolve into a severe hemorrhagic fever (severe dengue), which could lead to shock and death. Due to climate change, the disease is now threatening an increasing number of countries, with cases reported in Europe [2]. The existence of four different serotypes (DENV-1, 2, 3, 4), with also a fifth recently reported [3], complicates the development of an effective vaccine [4]. The four serotypes show not only significant differences in sequence similarity [5] but also distinctive infection dynamics. For example, DENV-1 is the most wide-spread serotype, followed by DENV-2 [6, 7], which is also more often associated with severe cases [8]. However, the mechanisms behind DENV infections and the complete set of differences between the serotypes are still unclear*.*

In severe cases, DENV can manifest as Viral Haemorrhagic Fever (VHF). The definition of VHF is complex since the symptoms can be mild or rare, but mainly caused by single-stranded RNA viruses from different families, such as flavivirus and filovirus [9]. However, not all the flaviviruses are associated with VHF, such as in the case of Zika virus (ZIKV), or the hemorrhagic symptoms can be secondary, for example intracranial hemorrhage causing paralysis or coma for Japanese Encephalitis virus (JEV [10];). In general, VHFs can be extremely dangerous in humans, as in the case of Ebola virus (EBOV). Other VHFs show not only similarities to severe dengue in terms of symptoms, but also in the transmission vector. For example, Yellow Fever virus (YFV) is also a mosquito-borne VHFs, with higher mortality but a slower rate of evolutionary change compared to DENV [11]. The mild VHF Chikungunya virus (CHIKV) shares the same vector with DENV, mosquito *Aedes aegypti*, and the two viruses can even coexist in the same mosquito [12]. However, while clinical and experimental analysis are the gold standard when comparing viruses, we still rely on sequence similarity approaches to understand the similarities between the thousands of available viral genomes.

The secondary structure of RNA viruses is fundamental for many viral functions, from encapsidation to egression from the cell and host defence [13–15]. Specific structures in the UTRs were found to be functional, for example, in DENV, but also in HIV and coronaviruses [13, 16, 17]. Other structural regions, including the 3′ UTR, were found conserved not only in DENV serotypes but also between DENV and ZIKV [18]. Moreover, the single-stranded RNA (ss-RNA) viruses preserve their structure (folding) even if their sequence mutates rapidly [19, 20]. Thus, the folding shows the potential to be used to classify different viral species and subspecies. ‘Selective 2′ Hydroxyl Acylation analyzed by Primer Extension’ (SHAPE) is a chemical-probing technique that uses different chemical agents (1 M7, NAI, NMIA) to bind single-stranded RNA regions in order to experimentally profile the RNA secondary structure. The technique was successfully applied to the complete genome of different viruses, including HIV-1, DENV, and recently SARS-CoV-2 [18, 21, 22].

However, while the RNA secondary structure is an informative element to characterise viruses, the secondary structure of only a few viral genomes has been experimentally characterized [18, 21]. Consequently, while thousands of viral genomes have been sequenced, we can only rely on in silico data to study their secondary structure. Furthermore, predicting the RNA secondary structure of entire viral genomes can be challenging, due to usually large sizes of > 10,000 nucleotides (nt), where most thermodynamic algorithms used to model the secondary structure drop in performance after 700 nt [23]. In our work, we computationally profiled the RNA secondary structure of > 7000 viral genomes (prioritising DENV serotypes and in general VHFs) using Computational Recognition of Secondary Structure (CROSS), a neural network trained on experimental genome-wide secondary structure profiling, including chemical-probing data, such as SHAPE, and enzyme-based, such as ‘Parallel Analysis of RNA Structure’ (PARS). The algorithm was successfully applied to predict HIV genome structure [24]. Furthermore, we mapped the secondary structure properties of the viruses on the world map, to study the genome interaction with proteins, and to further classify and understand the viruses.

## Results

### Structural properties of the DENV genomes

Here, we analysed the secondary structure profiles of the complete genomes of more than 7000 ss-RNA viruses (Table 1; Methods: Source of viral genomes). The structural profiles were generated using the CROSS algorithm, a fast and comprehensive alternative to profile the structural content (i.e., % of double-stranded nucleotides) of long and complex RNA molecules, such as viruses ([24]; see Methods: RNA secondary structure).

**Table 1:** The information regarding the number of genomes available, the family, and the average nucleotide length of each family for all the viruses used in our analysis. Hemorrhagic fevers marked with “^a^” shows symptoms only rarely or mild, while the ones with “^b^” are also reported as hemorrhagic diseases by WHO

Genome	Symbol	Family	Hemorrhagic	Number of genomes	Avg Size
Dengue 1 virus	DENV-1	Flavivirus	Yes^a^ (severe)	1634	10,500
Dengue 2 virus	DENV-2	Flavivirus	Yes^a^ (severe)	1184	9750
Dengue 3 virus	DENV-3	Flavivirus	Yes^a^ (severe)	772	10,590
Dengue 4 virus	DENV-4	Flavivirus	Yes^a^ (severe)	176	9730
Zika virus	ZIKV	Flavivirus	No	258	10,120
Chikungunya virus	CHIKV	Togavirus	Yes^a^ (mild)	522	10,500
Japanese encephalitis virus	JEV	Flavivirus	Yes^a^ (rare cases)	279	10,500
Yellow fever virus	YFV	Flavivirus	Yes	124	8530
West Nile fever virus	WNV	Flavivirus	Yes	1528	10,390
Tick-borne encephalitis virus	TBV	Flavivirus	Yes^b^	121	9770
Ebola virus	EBOV	Filovirus	Yes^b^	530	18,200

**Fig. 1:**
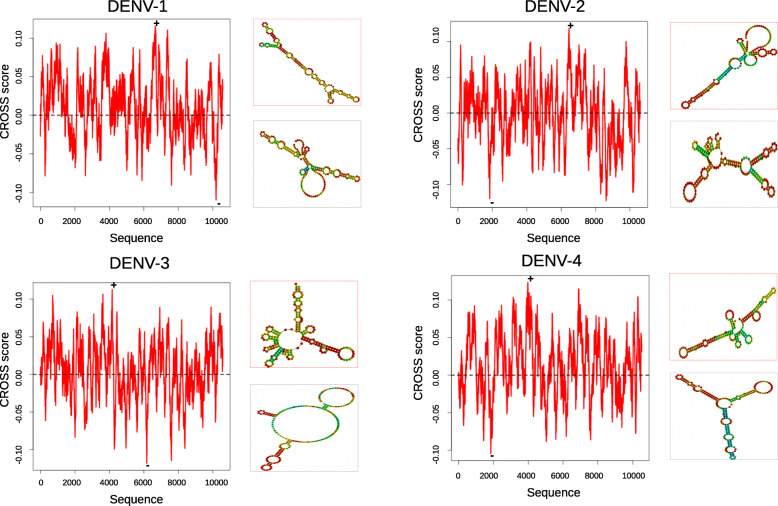
Secondary structure of the four DENV serotypes represented as propensity profiles. Nucleotides with a score > 0 are double-stranded, while < 0 indicates single-stranded nucleotides. The profile is normalised using the same formula reported in the original paper of CROSS methodology. For each profile, the highest (+) and lowest (−) structural peak is highlighted. The structures of 200 nt regions, including the most high-propensity double- (red) and single-stranded (gray) regions for each serotype, were computed using *RNAfold*

To analyse DENV secondary structure, we selected one strain for each serotype, focusing on strains that were widely used in previous publications [25]. In general, the four serotypes show significant differences in sequence, with around 65–70% sequence similarity [5]. Their secondary structure also shows notable differences (Fig. 1). For example, the 3′ UTR of DENV-1 shows a peculiar structural valley, compared to the others. Interestingly, DENV-1 and DENV-2 share the highest structural peak around 6000 nucleotides, while DENV-3 and DENV-4 also have the highest structural peak in common, but at position 4000.

**Fig. 2:**
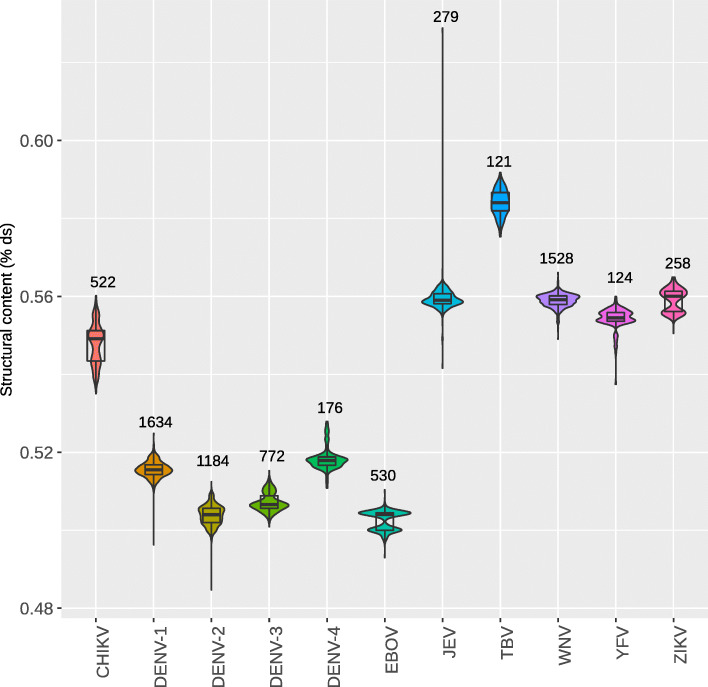
Structural content (% double-stranded nucleotides) for all the genomes for the 11 species. The number above each violin plot indicates the number of genomes used in each species

We further expanded our analysis to cover the 4 different serotypes of DENV, comprising ~ 4000 genomes (Fig. 2; Table 1). The analysis revealed that DENV-2 and DENV-3 are less structured than DENV-1 and DENV-4.

To confirm our approach, we compared our predictions with SHAPE experiments performed on DENV genomes [18]. Using the Area Under the ROC curve (AUC) to distinguish ranked SHAPE reactivities, we obtained an AUC ranging from 0.75 to 0.85 for DENV-2 and DENV-1 (Supplementary Figure 1a, b). This further supports the power of our in silico approach, which can generate thousands of secondary structure profiles with high performances on experimental data.

### Comparison of structural properties of the VHF genomes

Interestingly, DENV serotypes tend to be less structured than other flaviviruses, such as West Nile Fever virus (WNV), Yellow Fever virus (YFV), Tick-borne Encephalitis virus (TBV), and Japanese Encephalitis virus (JEV; Fig. 2). Even if not properly a VHF, we also used as comparison Zika virus (ZIKV), due to the similarities with DENV not only in the vector (*Aedes aegypti*), but also in terms of secondary structure domains [18]. Interestingly, while TEV and ZIKV genomes are more structured (average double-stranded nucleotides > 56%), WNF and JEV have a similar structural distribution, especially since they are also close in the species tree [26]. To further compare and classify the secondary structure of viral families outside of flavivirus, we also included > 500 genomes of EBOV, one of the most severe VHF, and CHIKV, exhibiting only mild and rare hemorrhagic symptoms but showing similarities with DENV and ZIKV in terms of vector and spreading (Fig. 2, Table 1). The analysis revealed that the other viruses are significantly more structured than DENV (mean structural content for Flaviviruses and DENV serotypes is 0.55, 0.51, respectively; Kolmogorov-Smirnov < 2.2e-16), with the exception of EBOV, which is predicted as one of the less structured (mean structural content 0.50).

### Structural properties of the terminal regions including untranslated regions of VHF genomes

**Fig. 3:**
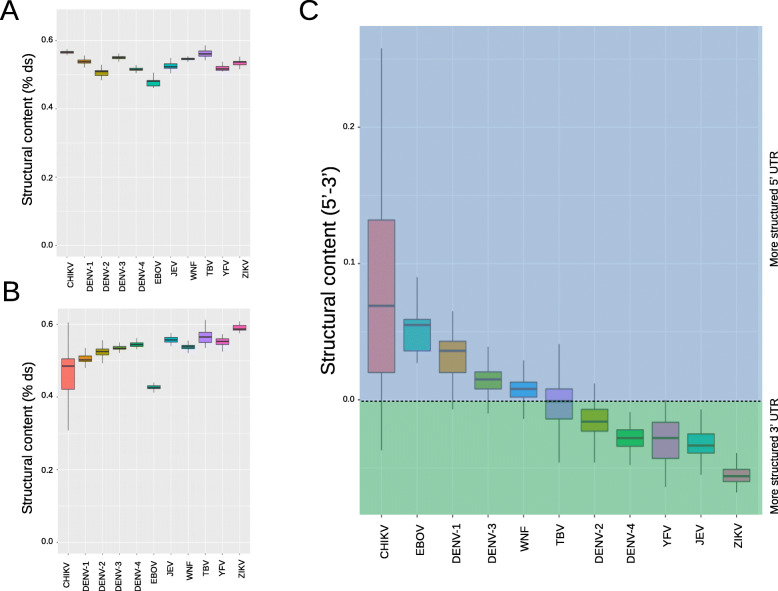
Structural content of the terminal regions including the UTRs of the 11 viral species. **a** Structural content (% double-stranded nucleotides) for all the genomes for the 11 species for the terminal region including the 5′ UTR. To have an equal comparison between the different species, we considered the 5′ UTR included in the first 1000 nt. The name used for the viruses is reported in Table 1. **b** Structural content (% double-stranded nucleotides) for all the genomes for the 11 species for the terminal region including the 3′ UTR. To have an equal comparison between the different species, we considered the 3′ UTR included in the last 1000 nt. **c** The difference for each individual genome between the structural content of the terminal regions including the 5′ and 3′ UTR. Viruses with more structured terminal region including the 5′ UTR are > 0 (blue area), while < 0 indicates more structured 3′ UTRs (green area)

To further study the secondary structure content for the > 7000 viral genomes, we also analysed the terminal regions including the 5′ and 3′ UTRs (first 1000 nt considered including the 5′ UTR; last 1000 nt considered including the 3′ UTR; Fig. 3a, b). Worth to specify that the terminal regions we are considering could have an overlap with coding genes, and this can go to less than 20% for TBV, up to 60% for ZIKV. DENV-3 is the only serotype with both terminal regions including UTRs more structured than the entire genome (5′ UTR = 0.55 and 3′ UTR = 0.53; Fig. 2), while DENV-1 has a more structured terminal region including the 5′ UTR (structural content = 0.53). The results are also consistent when considering only the UTRs (from ~ 70 to ~ 700 nt depending on the viral species; Supplementary Figure 2), highlighting a generally structured 3′ UTR for the flaviviruses, as expected for the presence of complex structures [27]. This result is in line with the experimental Parallel Analysis of RNA Structure (PARS) data coming from human RNAs, where the UTRs were more structured than the CDS [28]. This suggests that some viruses tend to mimic the secondary structure of human mRNAs to be efficiently translated by the cellular machinery [29]. This is also further supported in DENV, where a complex structure at the 3′ UTR was shown to mimic the absent polyA, to enhance translation [27]. Interestingly, EBOV has the least structured terminal regions including the UTRs (structural content 5′ UTR = 0.46; 3′ UTR = 0.41). In ZIKV, the terminal region including the 3′ UTR is more structured than the 5′ (Fig. 3c; 3′ UTR = 0.56, 5′ UTR = 0.50). CHIKV shows not only the highest structural variability in the terminal region including the 3′ UTR (standard deviation 3′ UTR = 0.16, Fig. 3b), with a more structured region at 5′ (3′ UTR = 0.43, 5′ UTR = 0.51; Fig. 3a). Finally, EBOV, DENV-1, and DENV-3 exhibit a more structured terminal region including the 5′ UTR, especially when compared with DENV-2, DENV-4 and JEV, which tend to be more structured (Fig. 3c).

### Structural content can be used to classify VHFs

**Fig. 4:**
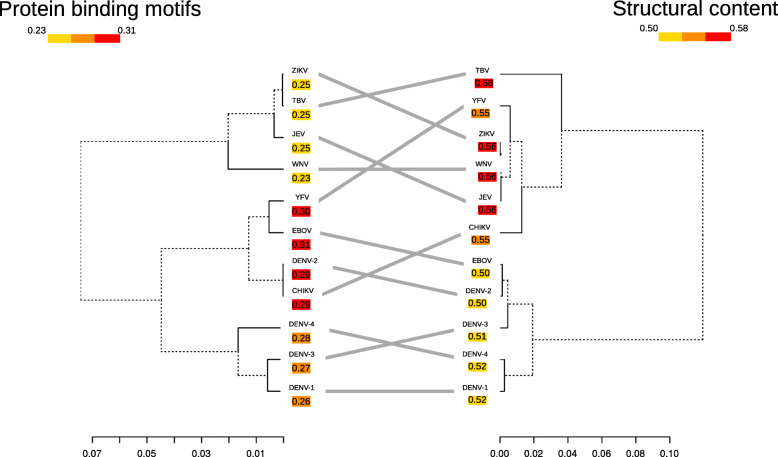
Comparison between the dendrograms obtained using the structural content (left side figure) and the number of binding motifs normalised by the length of the genome (right side figure). The gray lines connect the same virus species and serotypes. The abbreviations used for the viruses are explained in Table 1

The overall similarities and differences in structure are an additional feature that could be employed to characterise the different viruses. For the next step, the structural content (mean of the % of double-stranded nucleotides for all the viral genomes) in a specific species was used to hierarchically group the 11 different viruses (Methods: Hierarchical clustering; Table 1). The resulting dendrogram clustered the DENV serotypes, showing that they are more structurally similar compared to other viruses (Fig. 4). The structural similarities of DENV serotypes, together with the similarity between WNV and JEV, are in agreement with the Phylogenetic Tree of Viral Hemorrhagic Fevers [26].

The structural content revealed interesting clustering of the viruses. For example, while having a different genome sequence, DENV also clusters together with EBOV, since they share a less structured genome. According to its structural content, ZIKV is also part of the sub-cluster, together with WNV and JEV. The mosquito-transmitted YFV and CHIKV form a cluster, indicating that their structural content is similar. This is partially in agreement with the VHFs tree [26]. Interestingly, since TBV is more structured than any of these viruses, it forms an outlier. This is not a surprise, since it was previously shown that the secondary structure of mosquito- and tick-borne flaviviruses are more different, especially in the 3′ UTR [30]. To conclude, these results indicate that the level of secondary structure inside a viral genome can be used as a metric to build a tree of similarities, which could be further employed to classify viruses.

### Interaction between viral genomes and human host proteins can be used to classify VHFs

During translation and replication, ss-RNA viruses are naked RNA molecules inside human host cells. Previous studies already showed that genomes of the DENV interact with multiple human proteins during the infection and that the protein binding can enhance or inhibit the virulence [31]. Furthermore, RNA binding proteins tend to exhibit an altered activity during viral infection, in some cases due to the presence of highly abundant viral RNA, which can compete for the interaction with cellular RNA [32]. To study the relationship between human proteins and the viral RNA structures, we selected binding motifs from RNA Bind-n-Seq (RBNS) data of 78 human RNA-binding proteins [33], and searched the complete viral genomes for these motifs (Methods: Protein-RNA interactions). We observed that the 4 DENV serotypes have a different presence of protein binding domains, with DENV-2 showing the highest number of motifs, followed by DENV-4 (Supplementary Figure 3).

Similarly to the structural content analysis above, we used the number of protein binding domains to classify the viruses. To further understand how the connection between structure and interaction with proteins can classify viruses, we compared the resulting trees (Fig. 4). Interestingly, the DENV cluster is almost perfectly maintained, except that, for the number of protein interactions, DENV-2 is more similar to CHIKV than EBOV, which in turn is more related to YFV. Furthermore, clustering of WNV, JEV and ZIKV is partially maintained when using structure and interaction with proteins. To conclude, by analyzing thousands of different viral genomes, we identified specific clusters both in terms of secondary structure content and the potential number of interactions with proteins.

### Relationship between the structural content and number of protein interaction motifs

**Fig. 5:**
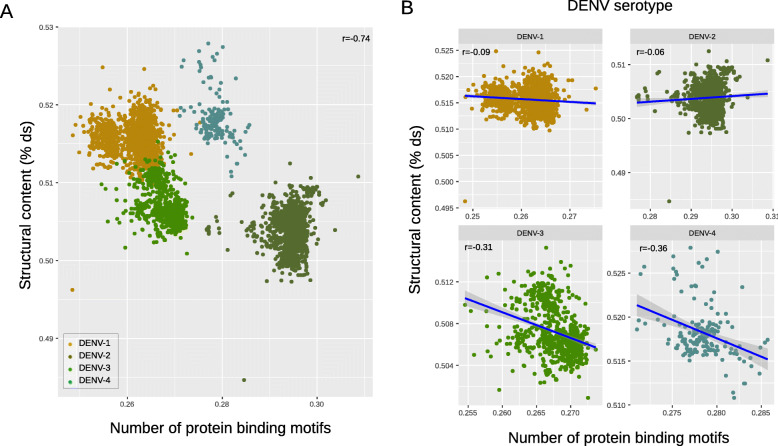
Correlation between the secondary structure and the interaction with proteins for all the DENV genomes. **a** Correlation between the structural content and the averaged number of binding domains for all the DENV genomes. **b** Correlation between the structural content and the averaged number of binding domains independently for the 4 DENV serotypes. The correlation is different when considering each serotype individually

Since both the structural content and the number of binding motifs can be used to classify the viruses, we hypothesized that there is a correlation between these two features. We found an overall high anti-correlation (r = − 0.74; *p*-value < 2.2e-16) between the number of protein-binding motifs and the structural content in DENV, meaning that less structured DENV genomes tend to bind more proteins (Fig. 5a). Interestingly, the different DENV serotypes cluster together according to their structure and the interaction with proteins (Fig. 5a). Also, the serotypes show a different trend when independently analysed, with DENV-3 and DENV-4 exhibiting the highest influence of the structure on the number of possible interacting proteins (r is − 0.31 and − 0.36; *p*-value < 2.2e-16 and 7.177e-07, respectively; Fig. 5b).

**Fig. 6:**
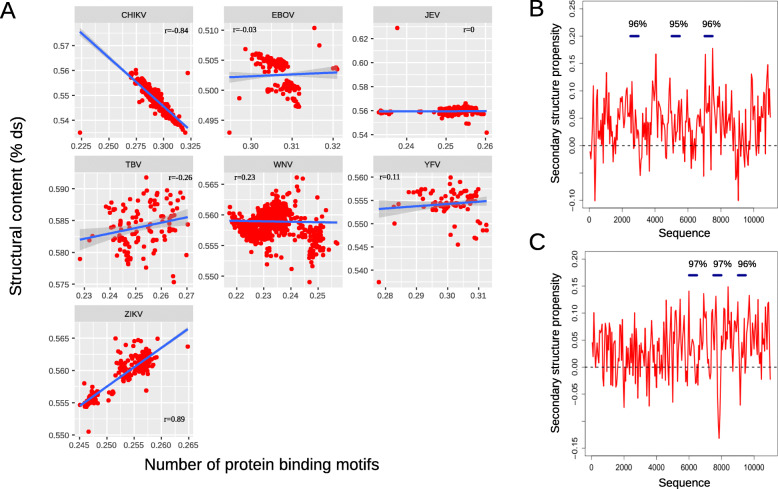
Correlation between the structural content and the averaged number of binding domains for the remaining 7 viral species. **a** Each point represents a single genome, while x- and y-axes indicate its structural content and the number of protein binding domains divided by the averaged size of the genome, respectively. **b** Consensus profile obtained from the secondary structure profiles of all the ZIKV genomes. Regions considered double-stranded for the majority of ZIKV genomes have a propensity > 0, while a score < 0 identifies consensus single-stranded regions. The blue bars mark the top 3 regions of 500 nt with the highest count of protein binding motifs. The percentage represents the average sequence conservation of the nucleotides inside that region. **c** Consensus profile obtained from the secondary structure profiles of all the CHIKV genomes. Regions considered double-stranded for the majority of CHIKV genomes have a propensity > 0, while a score < 0 identifies consensus single-stranded regions. The blue bars mark the top 3 regions of 500 nt with the highest count of protein binding motifs. The percentage represents the average sequence conservation of the nucleotides inside that region

Next, we compared the secondary structure and protein binding motifs of the other VHFs. The general picture is quite complex, with some viruses showing opposite trends between structure and interaction with proteins, providing a characteristic signature to further classify viruses into three categories. First, similarly to DENV, the mild hemorrhagic fever CHIKV shows a high anticorrelation (*r* = − 0.84; *p*-value < 2.2e-16, Fig. 6a), identifying a category of less structured but highly interactive viruses. DENV-3 and DENV-4 act similarly to CHIKV, having less structured genomes but highly interactive with proteins (Fig. 5b). Second, TBV and ZIKV show a positive correlation (Pearson correlation of 0.23 and 0.89; p-value 0.01 and < 2.2e-16 respectively; Fig. 6), identifying a category of high-structured viruses that are also highly interactive with host-proteins. Third, a class composed of JEV, WNV, and EBOV show almost no correlation between the structure and the possible interaction with proteins (r ~ 0). These results indicate a complex relationship between the structural content and protein-binding motifs, which can classify the viruses in 3 different categories: highly-structured and highly-interactive, poorly-structured and highly-interactive, or without a strong relationship between the overall structural content and interaction with proteins.

To further understand the different behaviour of ZIKV and CHIKV in terms of secondary structure and interaction with proteins, we generated a consensus-profile between the hundreds of genomes available (Methods; Fig. 6b, c). We also highlighted for each species the 3 regions in windows of 500 nt which have the highest-presence of protein binding motifs (Fig. 6b, c; blue bars). Interestingly, this analysis validates our overall observation, where the most contacted regions in ZIKV are very structured in the consensus profile obtained from all ZIKV genomes (Fig. 6b). Moreover, the most structured region from ZIKV consensus profile and one of the most highly-contacted by proteins is the one encoding for the nonstructural protein 3 (NS3), a helicase essential for viral replication [34]. We speculate that this region needs a very specific structure in order to be highly regulated by proteins. Conversely, the most contacted regions for CHIKV consensus secondary structure profile fall into highly unstructured regions (Fig. 6c). The least structured region of CHIKV consensus secondary structure profile, and one of the most regulated by proteins, encodes for the structural protein E3 [35]. Regardless of the structural propensity, the most interactive regions are also highly conserved in sequence both in ZIKV (~ 96% conservation) and slightly more in CHIKV (~ 97%) (Fig. 6b, c; Methods: Sequence similarity).

To extend and further validate this result, we checked the interactions between the most structured ZIKV and CHIKV viruses and > 1000 human RBPs. After selecting the 10 most and least structural genomes of ZIKV and CHIKV, we used the catRAPID algorithm [36] to predict > 4 × 10^6^ protein interactions between the genomes and human proteins (Methods: Protein-RNA interactions). Interestingly, the highly-structured ZIKV has stronger and more frequent interactions with proteins, reaching × 10 more strong interactions with proteins, compared to CHIKV (Supplementary Table 1). This result supports our hypothesis that ZIKV interacts with human proteins mainly using double-stranded regions when compared to CHIKV.

### Structural and protein interaction analysis of functionally related protein regions

We selected using NCBI annotations genomic regions encoding for proteins with a related function between the different viral families [37–39]. We selected the regions encoding for polymerases (Pol), helicases (Hel), and the structural protein hypothetically responsible for entering the human cells, which we called contact (Con), for the flaviviruses, filoviruses (EBOV), and togaviruses (CHIKV). The average structural content in the aforementioned regions highlight an interesting further classification (Supplementary Figure 4a). For example, the region associated with helicases (Hel) is more structured especially in flaviviruses, including DENV serotypes (DENV-1, DENV-2, DENV-4, WNV, TBV, YFV; Kolmogorov-Smirnov < 2.2e-16). Interestingly, the helicase region was also the most contacted region for ZIKV (Fig. 6b). Conversely, EBOV shows a significantly more structured Contact region (Con; Kolmogorov-Smirnov < 2.2e-16), associated to the GP protein, which plays a critical role in the host-cell entry process. To conclude, the structural content can further categorise viruses, as different genetic regions show a characteristic structural signature.

We further studied the amount of binding motifs and the structural content inside the regions corresponding to polymerase (Pol), helicase (Hel), and contact (Con) for each virus (Supplementary Figure 4b). Interestingly, the regions show an even further pattern that can be used for viral classification. The Hel region is highly-interactive with proteins in several flaviviruses (DENV-3, DENV-4, JEV, YFV, ZIKV; Kolmogorov-Smirnov < 2.2e-16). CHIKV is again an outlier, with a less interactive Hel region compared with Pol and Con.

**Fig. 7:**
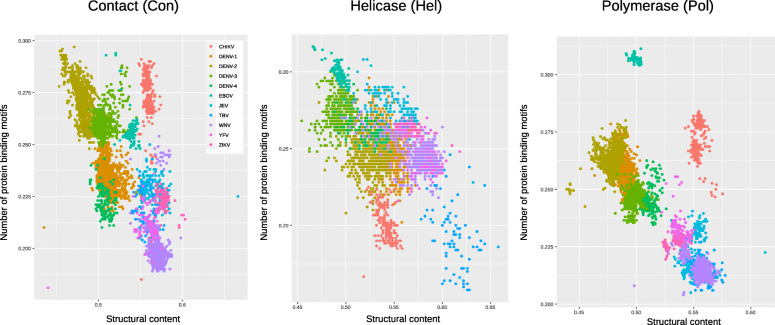
Clustering of protein viral regions according to their structural content and protein binding motifs. The following figure shows the correlation between Contact (Con), Helicase (Hel) and Polymerase (Pol) protein region for all the 11 viruses. The colors indicate the different viral families

An even stronger pattern emerges when clustering the viruses according to both interaction with proteins and secondary structure content (Fig. 7). The Pol region shows more aggregated clusters, especially for JEV, YFV, TBV, and WNV, while DENV serotypes, CHIKV and EBOV are the only one with a more defined cluster. This is in agreement with previous references, explaining how the polymerase tends to be more conserved in different viruses [38], following in our case a similar pattern of structure and regulation. While we did not observe clear clusters for the Hel region, probably due to the different protein families involved in this activity [37], the Con region shows more defined clusters for almost every virus.

### Geographical distribution of DENV serotypes

**Fig. 8:**
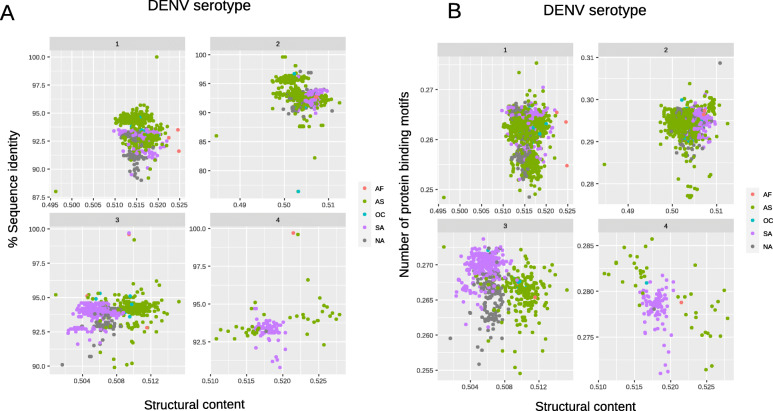
Correlation between the structural content and the averaged number of binding domains for the DENV serotypes, according to their geographical distribution. **a** Correlations between structural content and percentage of sequence identity. Samples coming from Africa (AF), Asia (AS), Oceania (OC), South America (SA), and North America (NA) were marked using different colours. Each point represents a single genome, while x- and y-axes indicate its structural content and the percentage of sequence identity. **b** Correlations between structural content and averaged number of binding domains. Samples coming from Africa (AF), Asia (AS), Oceania (OC), South America (SA), and North America (NA) were marked using different colours. Each point represents a single genome, while x- and y-axes indicate its structural content and the number of protein binding domains divided by the averaged size of the genome, respectively

We also studied the geographical connection between DENV serotypes and the secondary structure content. We found that the African DENV-1 is predicted to be more structured than the other serotypes, even when compared with the Asian strains (Supplementary Figure 5 Kolmogorov-Smirnov = 0.001), while on the contrary, Asian DENV-3 is more structured than the African strains (Kolmogorov-Smirnov = 0.07; Supplementary Figure 5). The structural content was also compared with pairwise sequence identity (Methods: Sequence Similarity) in order to identify the driving signal (Fig. 8a). Interestingly, DENV-3 is the one showing a more neat clustering of the Asian (AS) and South American (SA) strains. However, it is interesting to notice how the structural content is the feature driving the clustering, with the Asian and South American strain mainly lying on a sequence identity of ~ 93.5%.

Moreover, the correlation between binding motifs and secondary structure at the geographical level portrays a quite complex scenario (Fig. 8b). The clearest clusters are again identifiable for DENV-3, where the South-American strains are not only less structured, but also highly interacting with proteins. DENV-3 from South-America is also more distant from the Asian strains (Euclidean distance centroids × 1000 = 5), compared, for example, with DENV-1 (Euclidean distance centroids × 1000 = 1). Conversely, when comparing the protein binding motifs and sequence identity, the AS-SA cluster is disrupted, showing how the secondary structure information was essential for the identification of this cluster (Supplementary Figure 6). The analysis supports the hypothesis that secondary structure can be used to classify viruses and to discover possible differences identifiable or driven at geographic level.

## Discussion

The genomes of viral hemorrhagic fevers show different levels of secondary structure, especially structured in the UTR regions [16, 18]. This secondary structure is thought to be needed for different viral mechanisms, such as packaging and egression [13–15]. However, a comprehensive secondary structural landscape for their genomes was lacking. In our work, we computationally profiled and analysed the secondary structure profiles of more than 7000 complete viral genomes, including almost 4000 DENV samples, and 3500 other viral hemorrhagic fever-causing viruses. By studying the structural profiles, we observed that DENV is predicted to be less structured compared to viruses such as ZIKV, YFV, and WNV (Fig. 2). Conversely, DENV serotypes still tend to retain structured terminal regions including the UTRs, probably to be efficiently translated by the cellular machinery, similarly to human mRNAs [28, 40]. Single-stranded regions could be necessary to confer flexibility to the viral genomes, since flaviviruses need a high level of structural plasticity to undergo conformational changes during their life cycle, including circularization [41].

We also identified a correlation between the secondary structure and the number of protein binding domains, implying that the secondary structure is employed to regulate potential binding with proteins, as observed for human RNAs [42]. For viruses, the situation is more complex, as we observed a significantly positive (TBV and ZIKV) and negative (CHIKV, DENV-3, and DENV-4) relationship between secondary structure and the potential interaction with proteins. For example, ZIKV (positive RNA genome, positive correlation between secondary structure and interaction with proteins) and CHIKV (negative genome, negative correlation) belong to different viral families (Flavivirus and Togavirus respectively), and have a completely different capsid. Also while ZIKV shows a sequence similarity of 56% with YFV and JEV, it shows only 1.3% sequence identity with CHIKV [43]. Further investigation of additional viral families, especially togaviruses, are needed to elucidate the mechanism behind these opposing patterns.

Region encoding for viral helicases tend to be more structured in flaviviruses, while for other families, such as EBOV, contact protein regions show higher structural content. Moreover, region encoding for polymerases tend to be similarly structured across viruses and are potentially more bound by host-proteins, while the regions encoding for contact proteins often show species-specific clusters in terms of protein interactions and structure.

We also analysed the secondary structure at a geographical level, showing that DENV-3 strains from South-America and Asia have different patterns in their structure and the potential interaction with proteins, especially when compared with DENV-1 and DENV-2 (Fig. 8), and that the structure is a crucial elements for the identification of this cluster. Interestingly, this is in line with DENV-3 being the youngest serotype and the only one with a proposed origin not in Asia but in America [44]. This could explain the niched behaviour of DENV-3 in terms of structure and protein interactions, as well as supporting a possible independent origin of Asia and American DENV-3. Interestingly, DENV-4 shows a similar trend, but there are too few samples available to explain its evolution.

## Conclusions

In our study we employed secondary structural content and the presence of protein-binding domains to build similarity trees between VHFs in order to further characterise the viruses. The secondary structure and interaction with proteins can be used to cluster the viruses in agreement with previous phylogenetic trees, such as DENV serotypes, and JEV with WNV. Conversely, some relationships are surprising, as for example, DENV-2 is closer to EBOV when the secondary structure is used to establish similarity, but not when using the interaction with proteins. This result suggests how different measures, especially the secondary structure content, could be used to further classify and characterise different classes of viruses.

The inclusion of additional viral families or species, especially togaviruses and filoviruses, could further improve the analysis, providing even more data to explain some of the characteristic trends that we identified. Future experimental evidence, especially additional SHAPE profiles or cross-linking studies for the RBPs, will also help to extend and validate part of our analysis, as well as providing useful data to the scientific community.

Our massive computational analysis provided novel results regarding the secondary structure and the interaction with human proteins, not only for DENV serotypes but also for other viral hemorrhagic fevers. We envision that these approaches can be used by the scientific community to classify further and characterise these complex viruses.

## Methods

### Source of viral genomes

The viral genomes were downloaded from NCBI, selecting for each specific virus the fasta sequences containing the keyword *complete genome*. NCBI data was also used to extract the geographical information of DENV viruses as well as the serotypes. Fragmented or incomplete genomes were also removed. We also selected only complete genomes with only standard-nucleotides, filtering out sequences containing unknown (“N”) or degenerate (for example “R” or “Y”) nucleotides (Supplementary File 1).

### RNA secondary structure

The secondary structure profiles were computed using the CROSS (Computational Recognition of Secondary Structure) algorithm. CROSS is a neural network-based machine learning approach trained on experimental data (SHAPE, PARS, NMR/X-Ray, and icSHAPE), able to quickly profile large and complex molecules such as viral genomes without length restrictions. CROSS was already used to profile the complete HIV genome, also showing an AUC of 0.75 with experimental ‘Selective 2′ Hydroxyl Acylation analyzed by Primer Extension’ (SHAPE) data [24] and recently an AUC of 0.73 on SHAPE data for SARS-CoV-2 [45]. In the original manuscript, CROSS was also tested on crystallographic structures (AUC 0.72, PPV 0.74) and on DMS data for murine *XIST* (AUC 0.75) [24]. The algorithm was also updated and trained on structural in vivo data both with and without RNA methylation, and tested on the complete murine *XIST* [46]. For a comprehensive analysis, we used the *Global Score* model, considering nucleotides with a score > 0 as double-stranded, and < 0 as single-stranded. For the purpose of computing the structural content of a complete genome (i.e., % double-stranded nucleotides), the total number of nucleotides with a score > 0 was averaged for the total length of each genome. For the plots showing the complete secondary structure of DENV regions, we used the MFE structure computed using RNAfold [47].

### Protein-RNA interactions

To analyse the protein binding motifs in the viral genomes, we selected 5-mer motifs from the Table S3 of Dominguez et al. [33]. All of the 520 possible redundant motifs (270 non-redundant) were selected for further analysis. We scanned for the motifs on the complete genomes of the different strains, selecting only perfect matches. The number of motifs normalized by the average genome length of the different species of viruses was used to define a score for the number of potential interactions with proteins, according to the following formula:

$$ \frac{m}{avg(n)} $$

where m is the number of exact motifs found in a genome, and avg (n) is the average length of the genome of a specific species.

We also computed high-throughput predictions against the human proteome using the catRAPID Omics algorithm [36], which estimates the binding propensity between proteins and RNA by combining secondary structure, hydrogen bonding and van der Waals contributions. The algorithms computed more than 2 millions interactions between viral genomes and human proteins. The Discriminative Power (DP) was used to progressively filter for strong interactions. The Discriminative Power (DP) ranges from 0 to 1, where DP values above 0.5 indicate that the interaction is likely to take place.

### Hierarchical clustering

The structural content and the averaged number of binding domains were employed to build dendrograms. To this end, we computed the Euclidean distance between the values associated with each virus using statistical software R. We then used the *hclust* function based on a *ward. D2* module to build the dendrograms according to the hierarchical clustering.

### Secondary structure consensus profile

We used hundreds of profiles generated using CROSS to build secondary structure consensus profiles for ZIKV and CHIKV. To build the consensus profiles we selected non-overlapping windows of 50 nucleotides across all the genomes of each species, and we averaged CROSS propensity scores for each window in all the genomes available. To avoid problems due to the different lengths of the genomes, we limited the sliding window till the average length of the genomes of that specific species (Table 1). Regions with a score > 0 are double-stranded regions in agreement in all the genomes of a species, while < 0 for single-stranded consensus regions.

### Sequence similarity

To build pairwise sequence identities between the complete viral genomes we used the command line version of EMBOSS needle [48]. For a fast calculation we used a reference sequence for each dataset (same as used in Fig. 1), to align with all the sequences inside that specific dataset (for example all DENV-1 genomes). The algorithm was launched using standard parameters. The percentage of sequence identity was then used to identify the similarities in terms of primary structure (i.e. sequence) between the viruses. To extract conserved regions, we used a novel version of MAFFT [49] developed in April 2020, specifically built to perform multiple-alignments of huge viral genomes, such as the one of SARS-CoV-2. For each position in the alignment, we selected the nucleotide most present in that position, and we assigned the nucleotide and the associated percentage to a consensus profile. If a gap is identified as the most conserved, that position will have a value of 0.

## Supplementary Information

**Additional file 1: Supplementary Figure 1.** ROC curves of our predictions obtained using CROSS and experimental SHAPE data on DENV-2 (A) and DENV-1 (B). SHAPE data were ranked according to their reactivity, and the 5, 10 and 25% top/bottom nucleotides were selected. The AUC increases from 0.75 (25% top/bottom ranked SHAPE data; i.e. half of the dataset) to 0.85 (5% top/bottom ranked SHAPE data). Uncharacterised SHAPE reactivities < 0 were removed from the ranking. **Supplementary Figure 2.** Boxplot showing the structural content, as made for Fig. 3, but specifically selecting the 5′ and 3′ UTR. **Supplementary Figure 3.** Violin plot showing the interaction with proteins for each DENV serotype, computed as the presence of RNA binding motifs on their genome, averaged for the mean of the length of each serotype. **Supplementary Figure 4.** Boxplots showing for each virus how the regions coding for helicases (Hel), polymerases (Pol), and contact protein (Con) are different in terms of (A) structural content and (B) number of binding motifs. **Supplementary Figure 5.** Barplot showing for each DENV serotype the differences in structural content (% double-stranded nucleotides) in different geographical samples coming from Africa (AF), Asia (AS), Oceania (OC), South America (SA) and North America (NA). **Supplementary Figure 6.** Correlations between percentage of sequence identity and averaged number of binding domains. Samples coming from Africa (AF), Asia (AS), Oceania (OC), South America (SA), and North America (NA) were marked using different colours. Each point represents a single genome, while y- and x-axes indicate the percentage of sequence identity and the number of protein binding domains divided by the averaged size of the genome, respectively. **Supplementary Table 1.** Number of predicted interactions of all the human proteome and the 10 most structured ZIKV and CHIKV genomes. An increasing threshold on the Discriminative Power (DP) of catRAPID algorithm was used to iteratively select stronger interactions.

**Additional file 2.**

## Data Availability

All the data we used were available in NCBI (https://www.ncbi.nlm.nih.gov/) and downloaded as specified in the Methods on January/February 2020. Additional information and identifiers available in the Supplementary Files.

## Abbreviations

CROSS: Computational Recognition of Secondary Structure
SHAPE: Selective 2′ Hydroxyl Acylation analyzed by Primer Extension
PARS: Parallel Analysis of RNA Structure
VHF: Viral Haemorrhagic Fevers
DENV: Dengue virus
ZIKV: Zika virus
CHIKV: Chikungunya virus
WNV: West Nile Fever virus
JEV: Japanese Encephalitis virus
TBV: Tick-borne Encephalitis virus
EBOV: Ebola virus
YFV: Yellow Fever virus
RBP: RNA binding protein
UTR: Untranslated region
CDS: Coding sequence
AUC: Area under the ROC curve
ss-RNA: Single-stranded RNA
MFE: Minimum free energy
