# Supplementary material for: Structural landscape of the complete genomes of dengue virus serotypes and other viral hemorrhagic fevers
Source: BMC Genomics. 2021 May 17;22:352. doi: 10.1186/s12864-021-07638-7 (PMC8127238; doi:10.1186/s12864-021-07638-7)
Supplement: Supplementary file 1 — Additional file 1: Supplementary Figure 1. ROC curves of our predictions obtained using CROSS and experimental SHAPE data on DENV-2 (A) and DENV-1 (B). SHAPE data were ranked according to their reactivity, and the 5, 10 and 25% top/bottom nucleotides were selected. The AUC increases from 0.75 (25% top/bottom ranked SHAPE data; i.e. half of the dataset) to 0.85 (5% top/bottom ranked SHAPE data). Uncharacterised SHAPE reactivities < 0 were removed from the ranking. Supplementary Figure 2. Boxplot showing the structural content, as made for Fig. 3, but specifically selecting the 5′ and 3′ UTR. Supplementary Figure 3. Violin plot showing the interaction with proteins for each DENV serotype, computed as the presence of RNA binding motifs on their genome, averaged for the mean of the length of each serotype. Supplementary Figure 4. Boxplots showing for each virus how the regions coding for helicases (Hel), polymerases (Pol), and contact protein (Con) are different in terms of (A) structural content and (B) number of binding motifs. Supplementary Figure 5. Barplot showing for each DENV serotype the differences in structural content (% double-stranded nucleotides) in different geographical samples coming from Africa (AF), Asia (AS), Oceania (OC), South America (SA) and North America (NA). Supplementary Figure 6. Correlations between percentage of sequence identity and averaged number of binding domains. Samples coming from Africa (AF), Asia (AS), Oceania (OC), South America (SA), and North America (NA) were marked using different colours. Each point represents a single genome, while y- and x-axes indicate the percentage of sequence identity and the number of protein binding domains divided by the averaged size of the genome, respectively. Supplementary Table 1. Number of predicted interactions of all the human proteome and the 10 most structured ZIKV and CHIKV genomes. An increasing threshold on the Discriminative Power (DP) of catRAPID algorithm was used to iteratively sel [file 12864_2021_7638_MOESM1_ESM.pdf]

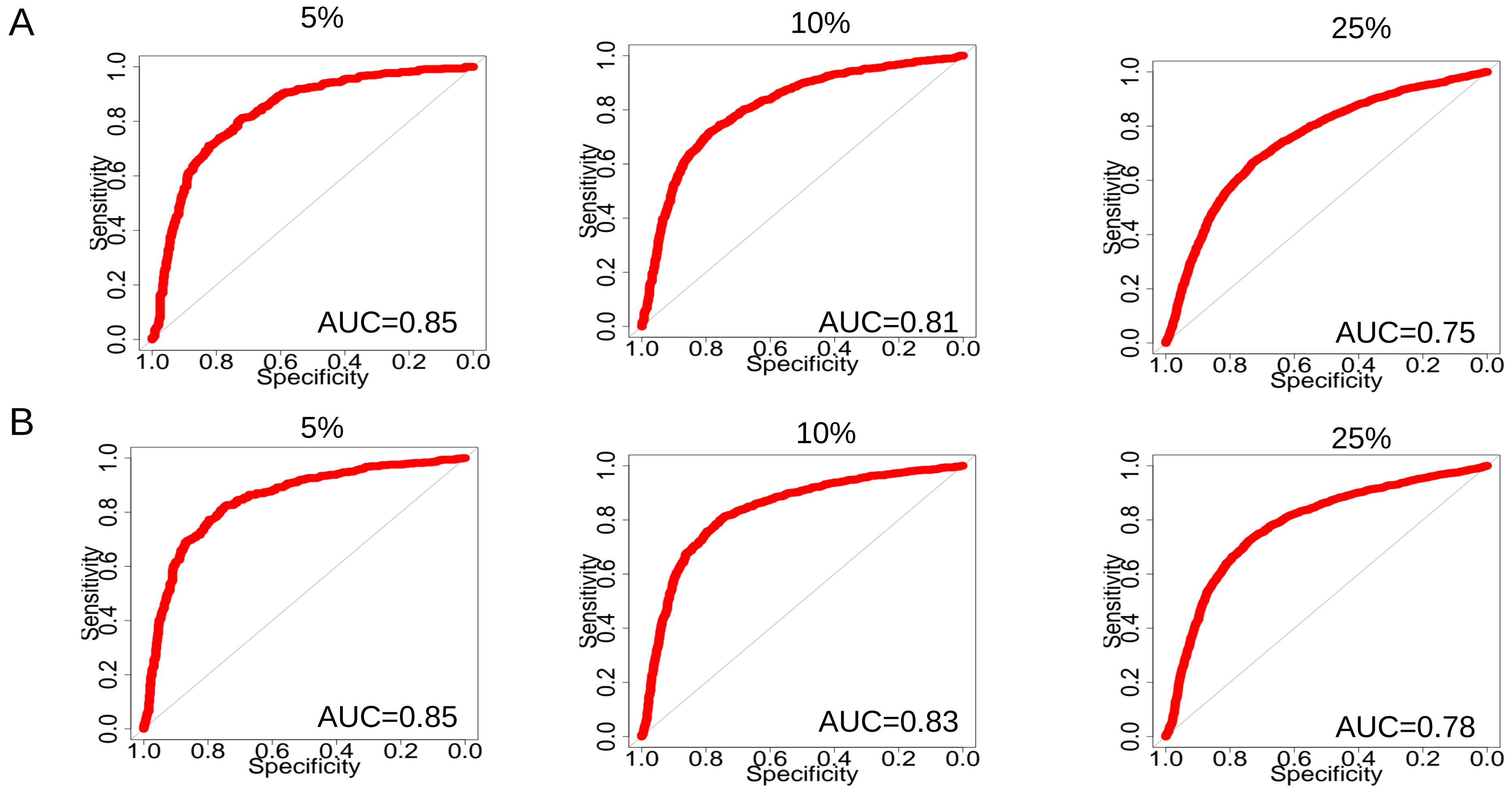

Supplementary Figure 1. ROC curves of our predictions obtained using CROSS and experimental SHAPE data on DENV-2 (A) and DENV-1 (B). SHAPE data were ranked according to their reactivity, and the 5%, 10% and 25% top/bottom nucleotides were selected. The AUC increases from 0.75 (25% top/bottom ranked SHAPE data; i.e. half of the dataset) to 0.85 (5% top/bottom ranked SHAPE data). Uncharacterised SHAPE reactivities <0 were removed from the ranking.

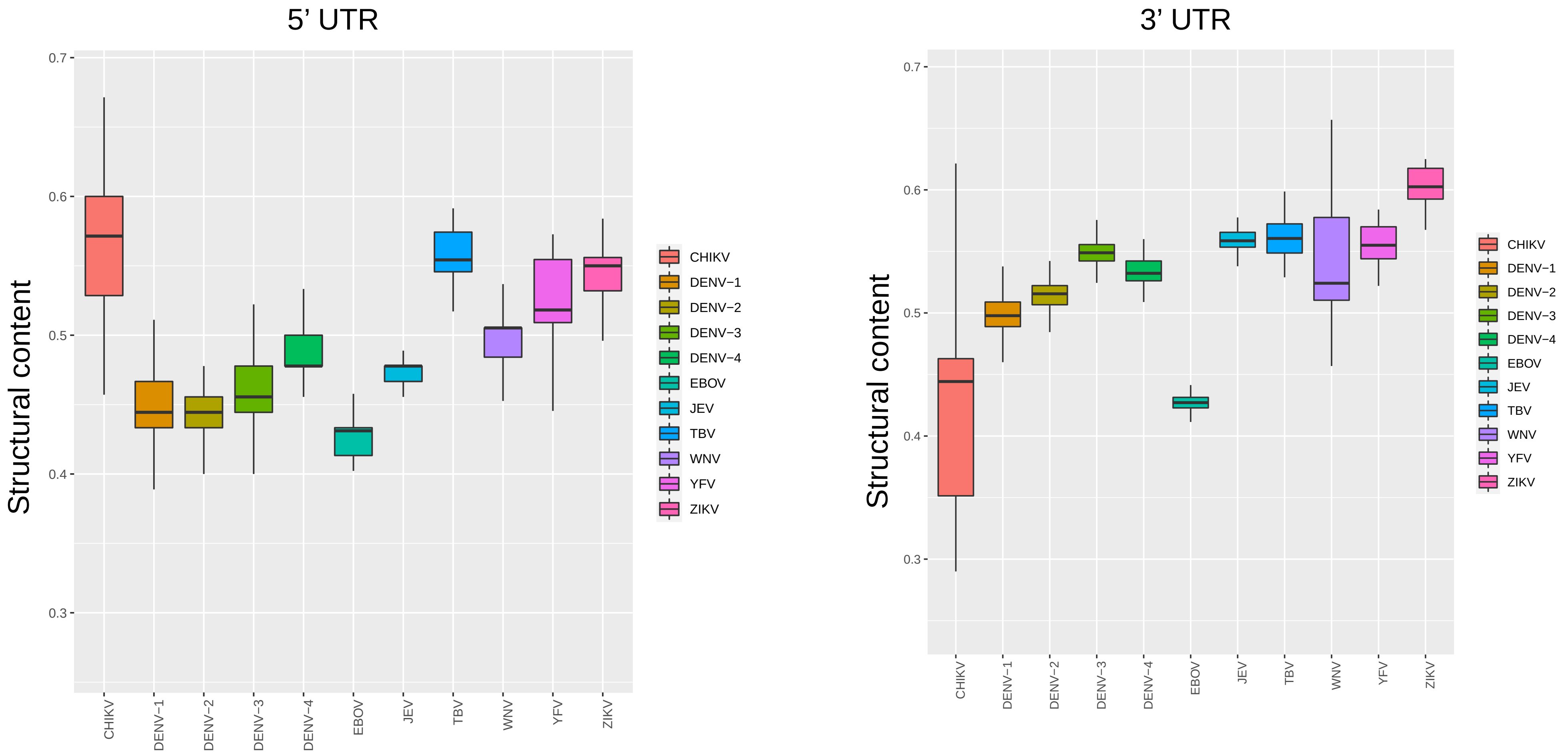

Supplementary Figure 2. Boxplot showing the structural content, as made for Figure 3, but specifically selecting the 5’ and 3’ UTR.

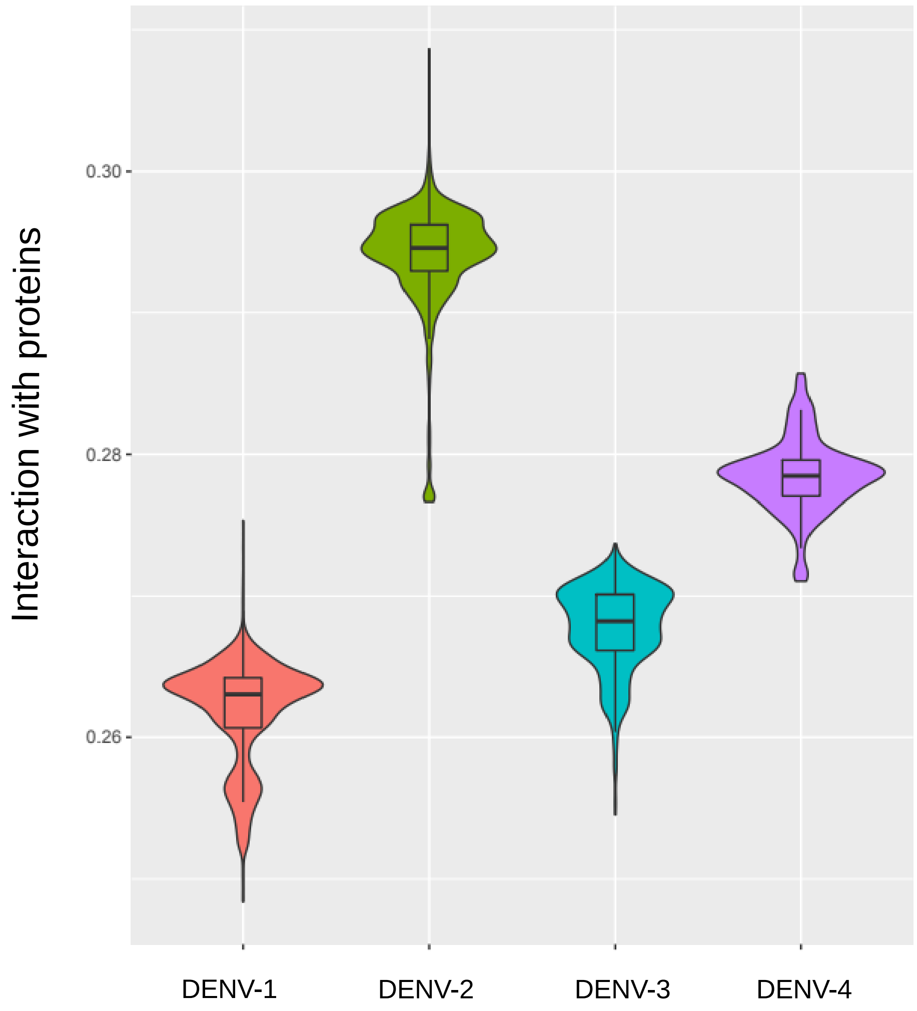

Supplementary Figure 3. Violin plot showing the interaction with proteins for each DENV serotype, computed as the presence of RNA binding motifs on their genome, averaged for the mean of the length of each serotype.

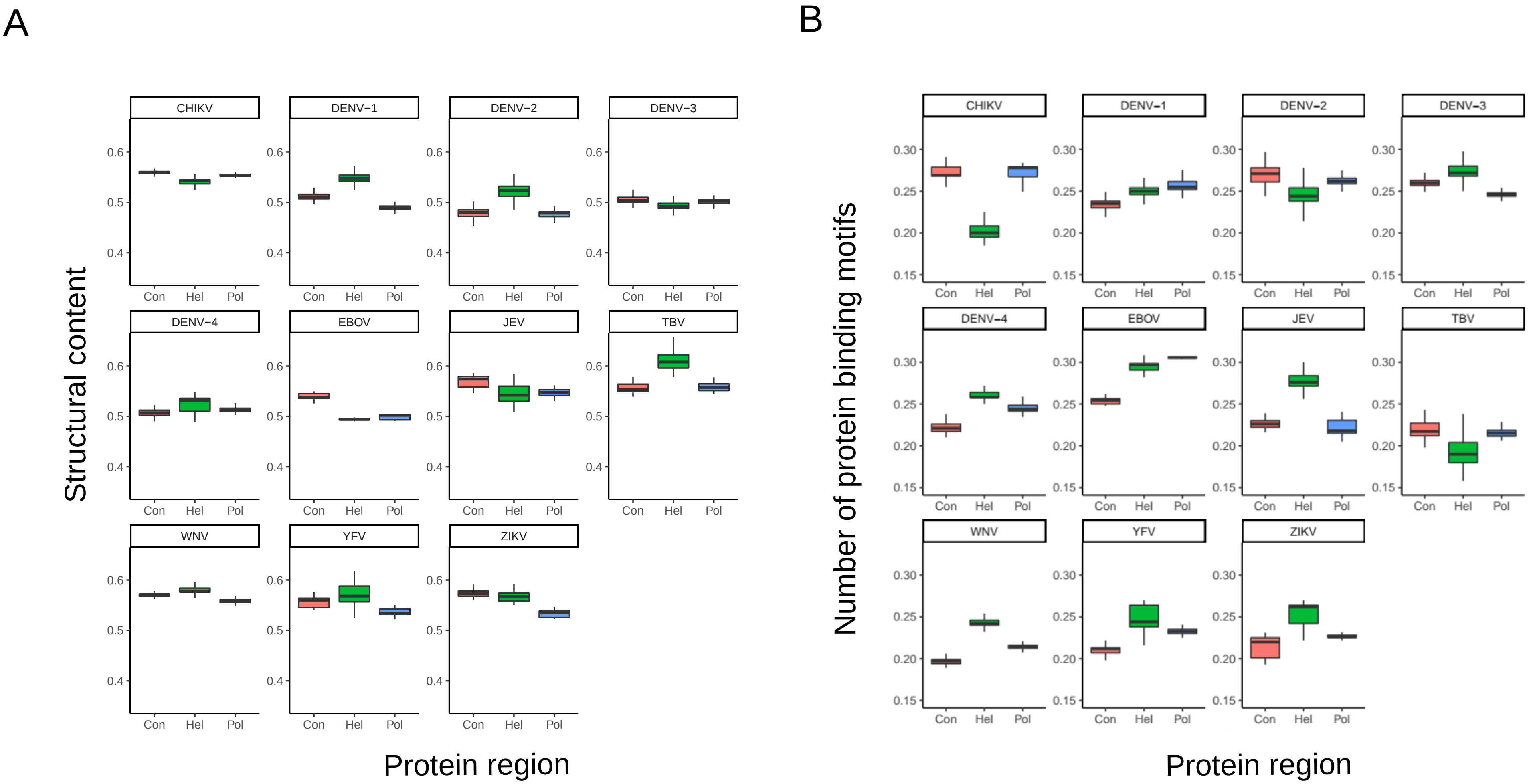

Supplementary Figure 4. Boxplots showing for each virus how the regions coding for helicases (Hel), polymerases (Pol), and contact protein (Con) are different in terms of (A) structural content and (B) number of binding motifs.

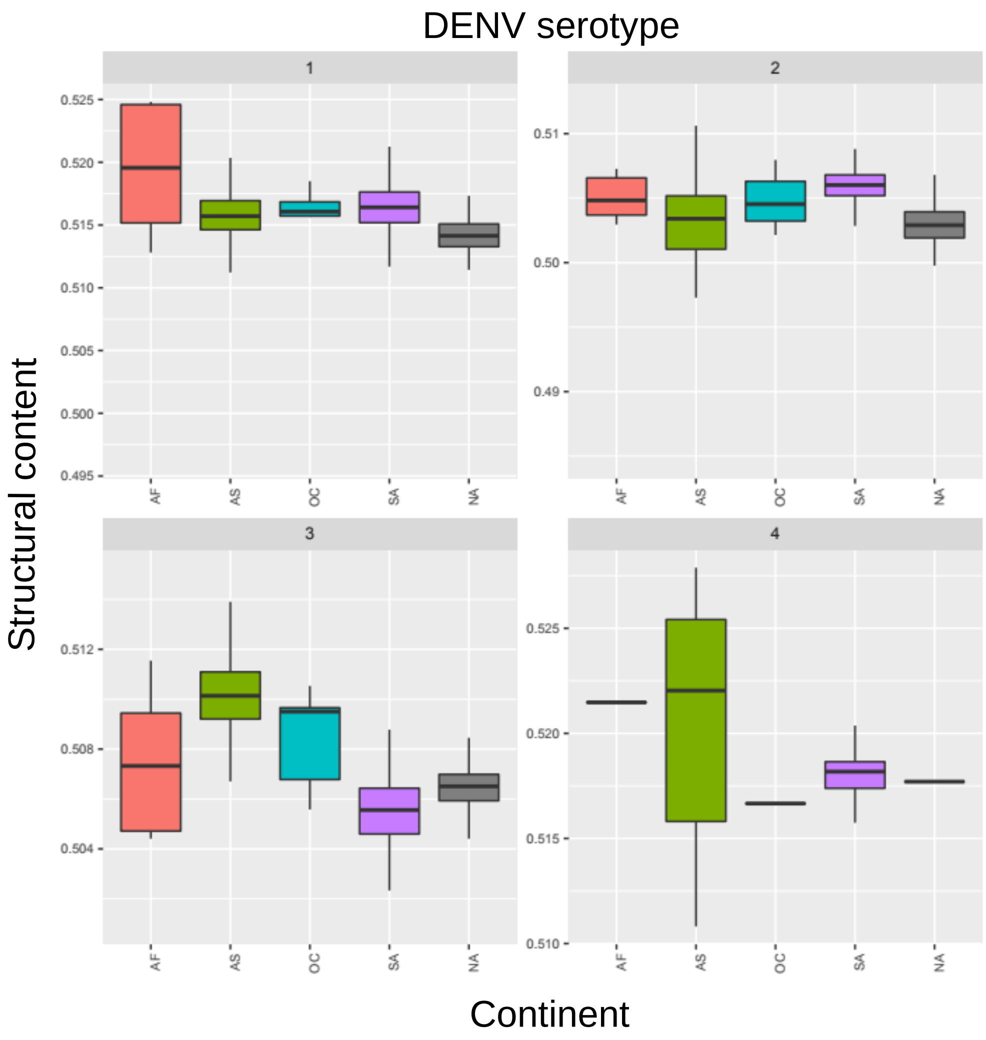

Supplementary Figure 5. Barplot showing for each DENV serotype the differences in structural content (% double-stranded nucleotides) in different geographical samples coming from Africa (AF), Asia (AS), Oceania (OC), South America (SA) and North America (NA).

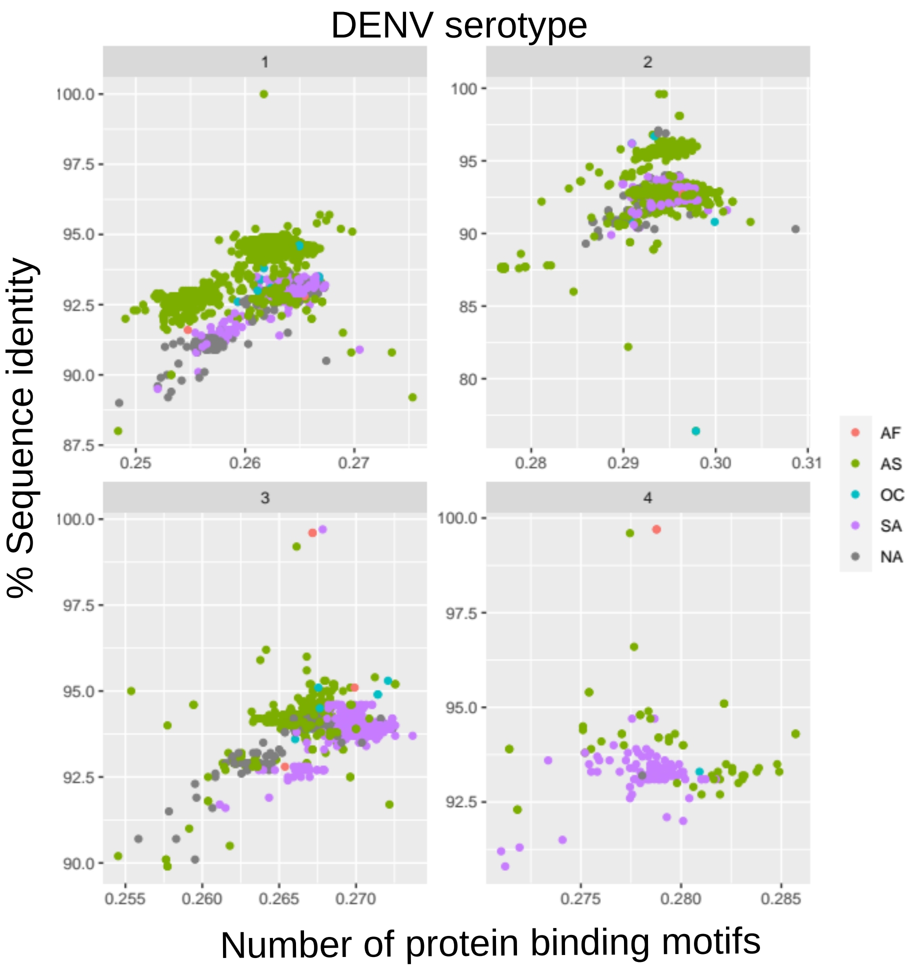

Supplementary Figure 6. Correlations between percentage of sequence identity and averaged number of binding domains. Samples coming from Africa (AF), Asia (AS), Oceania (OC), South America (SA), and North America (NA) were marked using different colours. Each point represents a single genome, while y- and x-axes indicate the percentage of sequence identity and the number of protein binding domains divided by the averaged size of the genome, respectively.

	DP>0.5	DP>0.7	DP>0.9
**ZIKV**	234’816 (x2.5)	80’017 (x4)	8’546 (x10)
**CHIKV**	90’909	19’056	773

Supplementary Table 1. Number of predicted interactions of all the human proteome and the 10 most structured ZIKV and CHIKV genomes. An increasing threshold on the Discriminative Power (DP) of catRAPID algorithm was used to iteratively select stronger interactions.
